# Supplementary material for: Clinical Outcomes and Prognostic Factors in Complex, High-Risk Indicated Procedure (CHIP) and High-Bleeding-Risk (HBR) Patients Undergoing Percutaneous Coronary Intervention with Sirolimus-Eluting Stent Implantation: 4-Year Results
Source: J Clin Med. 2023 Aug 15;12(16):5313. doi: 10.3390/jcm12165313 (PMC10455238; doi:10.3390/jcm12165313)
Supplement: Supplementary file 1 [file jcm-12-05313-s001.zip › jcm-2552054-supplementary.pdf]

Supplementary tables

Supplementary Table S1. Baseline characteristics: CHIP vs. non-CHIP.

Parameter	Non-CHIP N = 151	CHIP N = 81	P
Females	42 (28)	22 (27)	0.915
Age [years]	67 ± 11	70 ± 11	0.027
Acute coronary syndrome type at presentation			
UA	18 (11.9)	13 (16.0)	0.003
NSTEMI	10 (6.6)	16 (19.8)	
STEMI	19 (12.6)	13 (16.0)	
Cardiogenic shock	3 (2.0)	3 (3.7)	0.423
Arterial hypertension	130 (90.7)	76 (93.8)	0.412
Diabetes type 2	52 (34.4)	45 (55.6)	0.002
Dyslipidemia	107 (70.9)	70 (86.4)	0.008
Prior myocardial infarction	71 (47.0)	42 (51.9)	0.483
Prior PCI	82 (54.3)	48 (59.3)	0.469
Prior CABG	10 (6.6)	12 (14.8)	0.042
Chronic kidney disease	26 (17.2)	16 (19.8)	0.633
Prior stroke	11 (7.3)	6 (7.4)	0.973
Peripheral artery disease	13 (8.6)	12 (14.8)	0.146
COPD	7 (4.6)	6 (7.4)	0.385
Echocardiographic parameters			
LVEDd [mm]	49.4 ± 9.4	52.0 ± 7.9	0.179
IVSd [mm]	11.4 ± 2.0	11.4 ± 2.4	0.633
PWDd [mm]	10.4 ± 1.4	10.6 ± 1.9	0.331
LA [mm]	40.1 ± 5.8	41.0 ± 6.0	0.182
TAPSE [mm]	21.9 ± 4.2	22.2 ± 4.5	0.948
LVEF [%]	49.8 ± 10.6	48.9 ± 10.4	0.451
Severe MR	4 (3.3)	2 (2.7)	0.999
Severe AR	1 (0.8)	0	0.999
Severe AS	1 (0.8)	3 (4.1)	0.151

Supplementary Table S2. Baseline characteristics: HBR vs. non-HBR.

Parameter	Non-HBR<br>N = 156	HBR<br>N = 76	P
Females	39 (25)	25 (33%)	0.207
Age [years]	63 ± 9	77 ± 8	<0.001
Acute coronary syndrome type at presentation			
UA	20 (12.8)	11 (14.5)	0.271
NSTEMI	15 (9.6)	11 (14.5)	
STEMI	22 (14.1)	10 (13.2)	
Cardiogenic shock	3 (1.9)	3 (3.9)	0.396
Arterial hypertension	142 (91.0)	71 (93.4)	0.532
Diabetes type 2	53 (34.0)	44 (57.9)	<0.001
Dyslipidemia	116 (74.4)	61 (80.3)	0.321
Prior myocardial infarction	70 (44.9)	43 (56.6)	0.094
Prior PCI	81 (51.9)	49 (64.5)	0.071
Prior CABG	11 (7.1)	11 (14.5)	0.070
Chronic kidney disease	15 (9.6)	27 (35.5)	<0.001
Prior stroke	7 (4.5)	10 (13.2)	0.017
Peripheral artery disease	16 (10.3)	9 (11.8)	0.715
COPD	7 (4.5)	6 (7.9)	0.363
Echocardiographic parameters			
LVEDd [mm]	49.8 ± 9.2	51.5 ± 8.5	0.802
IVSd [mm]	11.4 ± 2.3	11.6 ± 1.7	0.422
PWDd [mm]	10.4 ± 1.6	10.5 ± 1.6	0.911
LA [mm]	38.9 ± 5.4	43.2 ± 5.8	<0.001
TAPSE [mm]	22.4 ± 4.1	21.3 ± 4.8	0.111
LVEF [%]	50.7 ± 9.3	47.0 ± 12.2	0.054
Severe MR	2 (1.6)	4 (6.1)	0.183
Severe AR	1 (0.8)	0	0.999
Severe AS	1 (0.8)	3 (4.5)	0.115

Supplementary Table S3. Laboratory test findings CHIP vs. non-CHIP.

Parameter	Non-CHIP<br>N = 151	CHIP<br>N = 81	P
White blood cells [10^9/L]	8.5 ± 2.9	8.6 ± 2.3	0.375
Hemoglobin [g/dL]	13.5 ± 1.6	13.1 ± 1.7	0.104
Red blood cells [10^{12}/L]	4.5 ± 0.5	4.3 ± 0.5	0.038
Platelets [10^9/L]	226.7 ± 65.9	215.8 ± 63	0.345
Glucose [md/dL]	124.2 ± 53.9	154.8 ± 75.3	0.001
HbA1c [%]	6.3 (5.8-7.2)	6.6 (6.1-7.3)	0.117
Total cholesterol [md/dL]	165.4 ± 44.0	161.5 ± 60.6	0.204
HDL [md/dL]	46.7 ± 15.8	44.0 ± 43.5	0.635
LDL [md/dL]	94.1 ± 38.7	83.0 ± 42.6	0.042
Triglycerides [md/dL]	124.3 ± 78.0	170.7 ± 89.9	0.112
Creatine [md/dL]	1.1 ± 0.6	1.2 ± 0.9	0.340
eGFR	72.2 ± 23.0	67.4 ± 23.4	0.211
TnI at admission [ng/mL]	75.5 (15.1-831.6)	211.5 (28.2-754.5)	0.292
Max. TnI [ng/mL]	1078 (42.0-8056.5)	1263 (88.2-13371)	0.358
CK	133 (84.5-310.5)	169 (75-319)	0.845
CK max	161.5 (93.8-498.5)	183 (80-390)	0.984
CK-MB	17 (14-26.2)	20 (13-34.5)	0.251
CK-MB max	20.5 (15-44.5)	26.5 (14.2-68)	0.338

Supplementary Table S4. Laboratory test findings HBR vs. non-HBR.

**Parameter**	**Non-HBR N = 156**	**HBR N = 76**	**P**
White blood cells [10^9/L]	8.8 ± 2.9	8.0 ± 2.2	0.041
Hemoglobin [g/dL]	14.0 ± 1.1	12.1 ± 1.9	<0.001
Red blood cells [10^{12}/L]	4.6 ± 0.4	4.1 ± 0.6	<0.001
Platelets [10^9/L]	223.1 ± 65.3	222.5 ± 64.7	0.816
Glucose [md/dL]	129.2 ± 55.1	148.3 ± 77.4	0.118
HbA1c [%]	6.3 (5.8-8.0)	6.4 (6.0-7.1)	0.572
Total cholesterol [md/dL]	177.3 ± 52.2	143.4 ± 41.4	<0.001
HDL [md/dL]	46.3 ± 15.5	44.7 ± 13.1	0.844
LDL [md/dL]	98.7 ± 41.0	76.0 ± 35.8	<0.001
Triglycerides [md/dL]	160.8 ± 62.1	113.1 ± 62.3	0.007
Creatine [md/dL]	1.0 ± 0.6	1.4 ± 0.8	<0.001
eGFR	77.5 ± 21.3	56.6 ± 20.4	<0.001
TnI at admission [ng/mL]	129 (15.3-197.2)	62.5 (19.0-1446)	0.711
Max. TnI [ng/mL]	1261 (60.0-11756.5)	372 (44.0-8802)	0.397
CK	164 (91.0-292)	118 (72-334)	0.147
CK max	180 (103-518.5)	156 (74-363)	0.152
CK-MB	17 (14-30)	18 (13-29.8)	0.921
CK-MB max	22 (15-61.2)	23.5 (14.2-40)	0.756

Supplementary Table S5. Lesion and procedure characteristics CHIP vs. non-CHIP.

**Parameter**	**Non-CHIP**<br>**N = 151**	**CHIP**<br>**N = 81**	**P**
Lesion location			
LM	1 (0.7)	8 (9.9)	0.0011
LAD	51 (33.8)	21 (25.9)	
LCx	45 (29.8)	16 (19.8)	
RCA	54 (35.8)	36 (44.4)	
VG	0	6 (7.4)	
Lesion type			
A	26 (17.2)	12 (14.8)	0.014
B1	49 (32.5)	17 (21)	
B2	28 (18.5)	9 (11.1)	
C	48 (31.8)	43 (53.1)	
Heavy calcification	9 (6.0)	9 (11.1)	0.162
Coronary bifurcation	11 (7.3)	12 (14.8)	0.067
SYNTAX	12.9 ± 8.7	16.0 ± 8.4	0.008
SYNTAX II PCI	31.6 ± 11.2	35.6 ± 10.1	0.003
SYNTAX II CABG	28.6 ± 11.1	29.9 ± 10.5	0.491
EuroScore II	1.3 (0.8 – 2.5)	2.5 (1.3 – 4.3)	<0.001
Lesion predilatation	96 (63.6)	47 (58.0)	0.407
Stent diameter [mm]	3.1 ± 0.5	3.3 ± 0.5	0.039
Stent length [mm]	18.3 ± 7.0	26.7 ± 14.3	<0.001
Stent pressure [atm]	15.2 (2.7)	15.5 ± 2.6	0.578
2nd stent	19 (12.7)	71 (87.7)	<0.001
Stent postdilatation	54 (35.8)	34 (42.0)	0.352
Access site			
Transradial	129 (85.4)	64 (79)	0.213
Transfemoral	24 (15.9)	19 (23.5)	
Guiding catheter			
6F	146 (96.7)	76 (93.8)	0.325
7F	6 (4.0)	5 (6.2)	
Dissection	5 (3.3)	11 (13.6)	0.003
MI typa 4a	4 (2.7)	1 (1.2)	0.660

Supplementary Table S6. Lesion and procedure characteristics HBR vs. non-HBR.

**Parameter**	**Non-HBR**<br>**N = 156**	**HBR**<br>**N = 76**	**P**
Lesion location			
LM	4 (2.6)	5 (6.6)	0.025
LAD	41 (26.3)	31 (40.8)	
LCx	47 (30.1)	14 (8.4)	
RCA	61 (39.1)	29 (38.2)	
VG	4 (2.6)	2 (2.6)	
Lesion type			
A	24 (15.4)	14 (18.4)	0.430
B1	42 (26.9)	24 (31.6)	
B2	29 (18.6)	8 (10.5)	
C	61 (39.1)	30 (39.5)	
Heavy calcification	12 (7.7)	6 (7.9)	0.957
Coronary bifurcation	17 (10.9)	6 (7.9)	0.473
SYNTAX	13.5 ± 8.4	14.8 ± 9.2	0.351
SYNTAX II PCI	29.6 ± 9.5	40.3 ± 10.5	<0.001
SYNTAX II CABG	29.5 ± 11.0	28.4 ± 10.5	0.514
EuroScore II	1.2 (0.8 – 2.3)	3.6 (1.8 – 6.6)	<0.001
Lesion predilatation	95 (60.9)	48 (63.2)	0.740
Stent diameter [mm]	3.2 ± 0.5	3.1 ± 0.5	0.472
Stent length [mm]	21.7 ± 11.8	20.3 ± 8.8	0.742
Stent pressure [atm]	15.3 ± 2.8	15.4 ± 2.5	0.452
2nd stent	56 (36.1)	34 (44.7)	0.207
Stent postdilatation	61 (39.1)	54 (35.8)	0.598
Access site			
Transradial	137 (87.8)	56 (73.7)	0.007
Transfemoral	23 (14.7)	20 (26.3)	
Guiding catheter			
6F	151 (96.8)	71 (93.4)	0.303
7F	6 (3.8)	5 (6.6)	
Dissection	13 (8.3)	3 (3.9)	0.216
MI typa 4a	4 (2.6)	1 (1.3)	0.999

Supplementary Table S7. Medications at discharge CHIP vs. non-CHIP.

**Parameter**	**Non-CHIP** N = 151	**CHIP** N = 81	**P**
Acetylsalicylic acid	151 (100)	81 (100)	1
P2Y12			
Clopidogrel	140 (92.7)	74 (91.4)	0.713
Prasugrel	0	1 (1.2)	0.349
Ticagrelor	11 (7.3)	6 (7.4)	0.973
Beta-blocker	143 (94.7)	80 (98.8)	0.167
Ca-blocker	32 (21.2)	21 (25.9)	0.413
Angiotensin converting enzyme inhibitor	124 (82.1)	66 (81.5)	0.904
Angiotensin receptor blocker	22 (14.6)	14 (17.3)	0.586
Diuretic	74 (49.0)	51 (63)	0.042
Mineralocorticoid receptor antagonist	32 (21.2)	16 (19.8)	0.796
Nitrates	5 (3.3)	8 (9.9)	0.068
Vitamin K antagonist	13 (8.6)	4 (4.9)	0.306
Novel oral anticoagulant	6 (3.9)	5 (6.1)	0.455
Statin	149 (98.7)	81 (100)	0.544
Hypoglycemic medications	33 (21.9)	29 (35.8)	0.022
Insulin	13 (8.6)	20 (24.7)	<0.001

Supplementary Table S8. Medications at discharge HBR vs. non-HBR.

**Parameter**	**Non-HBR**<br>**N = 156**	**HBR**<br>**N = 76**	**P**
Acetylsalicylic acid	156 (100)	76 (100)	1
P2Y12			
Clopidogrel	142 (91)	72 (94.7)	0.321
Prasugrel	1 (0.6)	0	0.99
Ticagrelor	13 (8.3)	4 (5.3)	0.400
Beta-blocker	150 (96.2)	73 (96.1)	0.999
Ca-blocker	41 (26.3)	12 (15.8)	0.074
Angiotensin converting enzyme inhibitor	130 (83.3)	60 (78.9)	0.415
Angiotensin receptor blocker	23 (14.7)	13 (17.1)	0.641
Diuretic	64 (41)	61 (80.3)	<0.001
Mineralocorticoid receptor antagonist	28 (17.9)	20 (26.3)	0.140
Nitrates	6 (3.8)	7 (9.2)	0.127
Vitamin K antagonist	1 (0.6)	16 (21.1)	<0.001
Novel oral anticoagulant	1 (0.6)	10 (13.1)	0.002
Statin	154 (98.7)	76 (100)	0.999
Hypoglycemic medications	39 (25)	23 (30.3)	0.395
Insulin	15 (9.6)	18 (23.7)	0.004

Supplementary Table S9. Univariable Cox regression for MACE: the whole population.

Characteristic	N	HR1	95% CI1	p-value
sex	232			
Female		—	—	
Male		1.06	0.53, 2.11	0.862
age	232	1.00	0.97, 1.03	0.969
CHIP	232	1.69	0.92, 3.10	0.090
HBR	232	1.84	1.00, 3.38	0.049
ACS	232	1.0	0.53, 1.85	0.987
LM	232	5.24	2.06, 13.4	<0.001
stent length	232	1.02	1.0, 1.04	0.126
stent diameter	232	1.37	0.75, 2.50	0.310
Lesion type	232			
A/B1		—	—	
B2/C		1.67	0.88, 3.17	0.119
calcification	232	2.67	1.19, 6.02	0.018
second stent	231	2.28	1.24, 4.19	0.008
MV predilat	232	1.59	0.81, 3.10	0.175
MV postdilat	232	1.75	0.95, 3.20	0.071
SYNTAX	212			
< 23		—	—	
23-33		1.80	0.74, 4.37	0.195
>= 33		1.11	0.15, 8.17	0.920
SYNTAX II PCI	210			
<= 21.5		—	—	
21.5-30.6		0.88	0.30, 2.63	0.821
>= 30.6		0.98	0.37, 2.64	0.976
SYNTAX II CABG	174			
<= 21.5		—	—	
21.5-30.6		0.68	0.26, 1.78	0.436
>= 30.6		1.03	0.45, 2.36	0.942
Euroscore II	232			
< 3		—	—	
3-5		1.90	0.86, 4.22	0.114
>= 5		2.78	1.29, 5.95	0.009
cardiogenic shock	232	2.25	0.54, 9.29	0.264
HT	232	1.92	0.46, 7.95	0.368
DM	232	1.85	1.01, 3.41	0.046
dyslipidemia	232	2.47	0.97, 6.28	0.058
MI	232	2.11	1.12, 3.96	0.021
PCI	232	2.13	1.09, 4.16	0.027
CABG	232	3.31	1.58, 6.93	0.001
AO	232	0.91	0.33, 2.55	0.859
Stroke	232	1.53	0.55, 4.29	0.419
Smoking	232	1.0	0.54, 1.82	0.986
CKD	232	1.72	0.86, 3.42	0.123
COPD	232	2.42	0.95, 6.16	0.064
medicine: clopidogrel	232	0.57	0.23, 1.46	0.245

Characteristic	N	HR1	95% CI1	p-value
medicine: ticagrelor	232	1.38	0.49, 3.85	0.544
medicine: ACEI	232	1.66	0.65, 4.23	0.285
medicine: ARB	232	0.57	0.20, 1.60	0.284
medicine: betablocker	232	0.78	0.19, 3.25	0.738
medicine: digoxin	232	2.74	0.37, 20.0	0.321
medicine: Ca-bloker	232	1.05	0.52, 2.14	0.887
medicine: diuretic	232	1.87	0.98, 3.55	0.056
medicine: MRA	232	1.40	0.70, 2.78	0.339
medicine: NTG	232	0.83	0.20, 3.44	0.797
medicine: alfa-adrenolitic	232	1.32	0.52, 3.37	0.558
medicine: ivabradine	232	6.57	0.90, 48.1	0.064
medicine: acenocumarol/warafarin	232	1.77	0.70, 4.51	0.230
medicine: rivaroxaban	232	2.28	0.70, 7.38	0.169
medicine: UFH	232	2.33	0.56, 9.66	0.243
medicine: IPP	232	1.55	0.69, 3.49	0.289
medicine: hypoglycaemic	232	1.79	0.96, 3.34	0.066
medicine: insulin	232	1.12	0.47, 2.65	0.801
echo: EF	195	0.98	0.96, 1.01	0.287
1HR = Hazard Ratio, CI = Confidence Interval				

Supplementary Table S10. Univariable Cox regression for TLR: the whole population.

**Characteristic**	**N**	**HR1**	**95% CI1**	**p-value**
sex	232			
Female		—	—	
Male		0.73	0.32, 1.63	0.437
age	232	0.99	0.95, 1.02	0.422
CHIP	232	1.76	0.82, 3.81	0.150
HBR	232	1.00	0.43, 2.29	0.991
ACS	232	1.68	0.71, 4.01	0.239
LM	232	5.13	1.54, 17.1	0.008
stent length	232	1.04	1.01, 1.07	0.005
stent diameter	232	1.58	0.73, 3.40	0.241
Lesion type	232			
A/B1		—	—	
B2/C		2.75	1.11, 6.86	0.030
calcification	232	3.29	1.24, 8.75	0.017
second stent	231	3.87	1.68, 8.90	0.001
MV predilat	232	2.72	1.03, 7.22	0.044
MV postdilat	232	2.00	0.93, 4.33	0.078
SYNTAX	212			
< 23		—	—	
23-33		1.39	0.41, 4.76	0.596
>= 33		1.73	0.23, 13.0	0.595
SYNTAX II PCI	210			
<= 21.5		—	—	
21.5-30.6		0.70	0.20, 2.50	0.587
>= 30.6		0.69	0.22, 2.18	0.530
SYNTAX II CABG	174			
<= 21.5		—	—	
21.5-30.6		0.45	0.11, 1.90	0.280
>= 30.6		1.34	0.46, 3.85	0.590
Euroscore II	232			
< 3		—	—	
3-5		1.33	0.45, 3.93	0.606
>= 5		1.81	0.61, 5.34	0.285
cardiogenic shock	232	1.83	0.25, 13.5	0.555
HTN	232	2.40	0.33, 17.7	0.391
DM	232	1.14	0.52, 2.47	0.750
dyslipidemia	232	2.55	0.77, 8.49	0.127
MI	232	2.19	0.98, 4.92	0.057
PCI	232	1.90	0.83, 4.38	0.129
CABG	232	3.08	1.16, 8.17	0.024
AO	232	0.72	0.17, 3.03	0.649
Stroke	232	2.03	0.61, 6.76	0.249
Smoking	232	0.90	0.42, 1.95	0.797
CKD	232	1.19	0.45, 3.16	0.727
COPD	232	1.40	0.33, 5.93	0.646

**Characteristic**	**N**	**HR1**	**95% CI1**	**p-value**
medicine: clopidogrel	232	0.64	0.19, 2.13	0.466
medicine: ticagrelor	232	1.01	0.24, 4.28	0.989
medicine: ACEI	232	5.44	0.74, 40.1	0.097
medicine: ARB	232	0.22	0.03, 1.61	0.136
medicine: betablocker	232	0.47	0.11, 2.00	0.308
medicine: Ca-blocker	232	1.48	0.64, 3.41	0.353
medicine: diuretic	232	1.30	0.60, 2.83	0.510
medicine: MRA	232	0.92	0.35, 2.45	0.873
medicine: NTG	232	0.66	0.09, 4.87	0.683
medicine: alfa-adrenolitic	232	1.77	0.61, 5.14	0.293
medicine: acenocumarol/warafarin	232	1.03	0.24, 4.36	0.967
medicine: rivaroxaban	232	1.18	0.16, 8.71	0.871
medicine: IPP	232	1.32	0.50, 3.50	0.577
medicine: hypoglycaemic	232	1.05	0.44, 2.50	0.907
medicine: insulin	232	0.57	0.13, 2.40	0.441
echo: EF	195	1.03	0.98, 1.08	0.244
1HR = Hazard Ratio, CI = Confidence Interval				

Supplementary Table S11. Univariable Cox regression for MACE: CHIP population.

**Characteristic**	**N**	**HR1**	**95% CI1**	**p-value**
Sex	81			
Female		—	—	
Male		0.77	0.29, 2.02	0.590
Age	81	1.01	0.97, 1.05	0.648
HBR	81	1.97	0.79, 4.91	0.143
ACS	81	1.17	0.48, 2.89	0.730
LM	81	3.33	1.10, 10.1	0.033
stent_length	81	1.01	0.98, 1.04	0.579
stent_diameter	81	1.25	0.51, 3.04	0.624
Lesion type	81			
A/B1		—	—	
B2/C		1.50	0.54, 4.16	0.438
calcification	81	2.27	0.75, 6.86	0.146
second_stent	81	0.74	0.22, 2.55	0.638
MV_predilat	81	1.55	0.59, 4.08	0.374
MV_postdilat	81	1.60	0.65, 3.93	0.308
SYNTAX	70			
< 23		—	—	
23-33		2.26	0.71, 7.21	0.169
>= 33		0.00	0.00, Inf	0.998
SYNTAX_II_PCI	69			
<= 21.5		—	—	
21.5-30.6		0.41	0.04, 3.95	0.441
>= 30.6		0.54	0.07, 4.22	0.557
SYNTAX_II_CABG	61			
<= 21.5		—	—	
21.5-30.6		3.54	0.43, 29.4	0.242
>= 30.6		3.79	0.47, 30.3	0.209
Euroscore_II	81			
< 3		—	—	

Characteristic	N	HR1	95% CI1	p-value
3-5		2.32	0.80, 6.70	0.119
>= 5		2.55	0.83, 7.80	0.101
cardiogenic_shock	81	1.78	0.24, 13.3	0.575
HT	81	1.33	0.18, 9.99	0.779
DM	81	2.51	0.90, 6.96	0.078
dyslipidemia	81	3.08	0.41, 23.1	0.273
MI	81	3.06	1.10, 8.51	0.032
PCI	81	2.86	0.95, 8.63	0.062
CABG	81	2.48	0.89, 6.90	0.081
AO	81	1.15	0.34, 3.96	0.821
Stroke	81	2.02	0.47, 8.76	0.348
Smoking	81	0.45	0.18, 1.12	0.084
Chronic kidney disease	81	2.79	1.10, 7.09	0.031
COPD	81	1.28	0.30, 5.54	0.743
medicine_clopidogrel	81	0.43	0.13, 1.48	0.182
medicine_ticagrelor	81	1.53	0.35, 6.65	0.568
medicine_ACEI	81	1.16	0.34, 3.97	0.818
medicine_ARB	81	0.96	0.28, 3.29	0.947
medicine_betablocker	81	0.01	0.00, 0.20	0.002
medicine_Ca_blocker	81	0.78	0.26, 2.34	0.655
medicine_diuretic	81	3.64	1.06, 12.5	0.040
medicine_MRA	81	1.94	0.74, 5.10	0.180
medicine_alfa_adrenolitic	81	2.22	0.51, 9.63	0.287
medicine_ivabradine	81	4.66	0.61, 35.3	0.136
medicine_acenocumarol_warafarin	81	2.33	0.54, 10.1	0.258
medicine_rivaroxaban	81	4.24	1.23, 14.6	0.022
medicine_IPP	81	1.76	0.41, 7.63	0.449
medicine_hypoglycaemic	81	2.00	0.81, 4.91	0.133
medicine_insulin	81	0.86	0.29, 2.59	0.787
echo_EF	74	0.97	0.94, 1.01	0.168
1HR = Hazard Ratio, CI = Confidence Interval				

Supplementary Table S12. Univariable Cox regression for TLR: CHIP population.

**Characteristic**	**N**	**HR1**	**95% CI1**	**p-value**
Sex	81			
Female		—	—	
Male		0.50	0.16, 1.58	0.239
Age	81	0.99	0.95, 1.04	0.763
HBR	81	0.72	0.22, 2.38	0.587
ACS	81	1.04	0.34, 3.24	0.939
LM	81	2.46	0.54, 11.2	0.246
stent_length	81	1.03	0.99, 1.07	0.101
stent_diameter	81	1.87	0.63, 5.62	0.262
Lesion type	81			
A/B1		—	—	
B2/C		5.82	0.75, 45.1	0.092
calcification	81	2.71	0.73, 10.1	0.135
second_stent	81	0.70	0.15, 3.18	0.641
MV_predilat	81	3.53	0.77, 16.1	0.103
MV_postdilat	81	2.83	0.85, 9.40	0.090
SYNTAX	70			
< 23		—	—	
23-33		1.83	0.37, 9.09	0.459
>= 33		0.00	0.00, Inf	0.999
SYNTAX_II_PCI	69			
<= 21.5		—	—	
21.5-30.6		0.28	0.03, 3.10	0.300
>= 30.6		0.28	0.03, 2.37	0.240
SYNTAX_II_CABG	61			
<= 21.5		—	—	
21.5-30.6		1.73	0.18, 16.7	0.633
>= 30.6		2.78	0.34, 23.1	0.343
Euroscore_II	81			
< 3		—	—	

Characteristic	N	HR1	95% CI1	p-value
3-5		1.54	0.38, 6.16	0.543
>= 5		2.03	0.51, 8.12	0.317
cardiogenic_shock	81	3.00	0.39, 23.2	0.293
HT	81	0.82	0.11, 6.38	0.853
DM	81	1.77	0.53, 5.89	0.350
dyslipidemia	81	1.88	0.24, 14.6	0.545
MI	81	2.17	0.65, 7.21	0.207
PCI	81	1.51	0.45, 5.02	0.501
CABG	81	3.43	1.03, 11.4	0.044
AO	81	0.56	0.07, 4.31	0.575
stroke	81	3.36	0.73, 15.4	0.119
Smoking	81	0.45	0.14, 1.42	0.175
CKD	81	1.56	0.42, 5.79	0.503
medicine_clopidogrel	81	0.25	0.07, 0.94	0.039
medicine_ticagrelor	81	2.52	0.55, 11.5	0.233
medicine_ACEI	81	2.40	0.31, 18.6	0.402
medicine_ARB	81	0.46	0.06, 3.57	0.458
medicine_Ca_blocker	81	0.96	0.26, 3.54	0.948
medicine_diuretic	81	2.01	0.54, 7.42	0.296
medicine_MRA	81	0.83	0.18, 3.80	0.814
medicine_alfa_adrenolitic	81	1.69	0.22, 13.1	0.617
medicine_acenocumarol_warafarin	81	1.76	0.23, 13.6	0.590
medicine_rivaroxaban	81	1.94	0.25, 15.1	0.526
medicine_IPP	81	1.03	0.22, 4.68	0.974
medicine_hypoglycaemic	81	1.25	0.40, 3.95	0.700
medicine_insulin	81	0.30	0.04, 2.31	0.248
echo_EF	74	1.05	0.97, 1.13	0.249
1HR = Hazard Ratio, CI = Confidence Interval				

Supplementary Table S13. Univariable Cox regression for MACE: HBR population.

**Characteristic**	**N**	**HR[1]**	**95% CI[1]**	**p-value**
Sex	76			
Female		—	—	
Male		0.79	0.31, 2.00	0.612
Age	76	0.97	0.92, 1.02	0.187
CHIP	76	1.78	0.72, 4.44	0.213
ACS	76	0.72	0.29, 1.77	0.476
LM	76	3.71	1.08, 12.7	0.038
stent_length	76	0.99	0.94, 1.04	0.662
stent_diameter	76	0.63	0.23, 1.68	0.354
Lesion type	76			
A/B1		—	—	
B2/C		0.87	0.35, 2.15	0.765
calcification	76	2.71	0.79, 9.32	0.115
second_stent	76	1.92	0.77, 4.78	0.159
MV_predilat	76	1.70	0.61, 4.72	0.308
MV_postdilat	76	1.76	0.72, 4.34	0.217
SYNTAX	66			
< 23		—	—	
23-33		3.27	0.91, 11.8	0.070
>= 33		0.00	0.00, Inf	0.998
SYNTAX_II_CABG	63			
<= 21.5		—	—	
21.5-30.6		0.61	0.16, 2.28	0.466
>= 30.6		1.01	0.32, 3.18	0.986
Euroscore_II	76			
< 3		—	—	
3-5		3.20	0.92, 11.1	0.067
>= 5		3.43	1.15, 10.2	0.027
cardiogenic_shock	76	4.75	1.09, 20.7	0.038
HT	76	1.34	0.18, 10.1	0.775
DM	76	3.36	1.11, 10.1	0.032
dyslipidemia	76	5.20	0.69, 39.0	0.108
MI	76	2.35	0.85, 6.52	0.102
PCI	76	2.13	0.71, 6.43	0.178
CABG	76	2.45	0.88, 6.80	0.086
AO	76	2.29	0.76, 6.91	0.142
stroke	76	0.90	0.21, 3.92	0.892
smoking	76	2.47	0.99, 6.14	0.052
CKD	76	2.90	1.17, 7.23	0.022
COPD	76	3.55	1.17, 10.7	0.025
medicine_clopidogrel	76	0.99	0.13, 7.44	0.995
medicine_ticagrelor	76	1.01	0.13, 7.54	0.995
medicine_ACEI	76	1.47	0.43, 5.04	0.542
medicine_ARB	76	0.56	0.13, 2.41	0.435
medicine_betablocker	76	0.60	0.08, 4.51	0.620

Characteristic	N	HR[1]	95% CI[1]	p-value
medicine_digoxin	76	2.37	0.31, 17.8	0.402
medicine_Ca_blocker	76	1.50	0.50, 4.54	0.469
medicine_diuretic	76	2.40	0.55, 10.4	0.242
medicine_MRA	76	2.22	0.89, 5.53	0.086
medicine_NTG	76	0.56	0.08, 4.23	0.578
medicine_alfa_adrenolitic	76	1.82	0.60, 5.50	0.287
medicine_ivabradine	76	4.37	0.58, 33.1	0.153
medicine_acenocumarol_warafarin	76	1.31	0.47, 3.65	0.600
medicine_rivaroxaban	76	1.10	0.25, 4.78	0.896
medicine_UFH	76	1.62	0.37, 7.01	0.521
medicine_IPP	76	1.07	0.31, 3.67	0.914
medicine_hypoglycaemic	76	4.03	1.62, 10.1	0.003
medicine_insulin	76	1.35	0.49, 3.75	0.565
echo_EF	67	0.97	0.94, 1.01	0.113

[1]HR = Hazard Ratio, CI = Confidence Interval

Supplementary Table S14. Univariable Cox regression for TLR: HBR population.

**Characteristic**	**N**	**HR[1]**	**95% CI[1]**	**p-value**
Sex	76			
Female		—	—	
Male		0.29	0.07, 1.22	0.092
Age	76	0.96	0.89, 1.03	0.267
CHIP	76	1.28	0.32, 5.13	0.726
ACS	76	1.06	0.25, 4.43	0.937
LM	76	2.79	0.34, 22.7	0.337
stent_length	76	1.02	0.95, 1.10	0.503
stent_diameter	76	0.32	0.05, 1.99	0.222
Lesion type	76			
A/B1		—	—	
B2/C		1.60	0.38, 6.69	0.520
calcification	76	1.95	0.24, 16.0	0.532
second_stent	76	2.27	0.54, 9.51	0.261
MV_postdilat	76	5.73	1.16, 28.4	0.033
SYNTAX	66			
< 23		—	—	
23-33		2.32	0.27, 20.0	0.445
>= 33		0.00	0.00, Inf	0.999
SYNTAX_II_CABG	63			
<= 21.5		—	—	
21.5-30.6		0.00	0.00, Inf	0.998
>= 30.6		1.19	0.28, 4.97	0.815
Euroscore_II	76			
< 3		—	—	
3-5		3.09	0.43, 22.0	0.260
>= 5		3.92	0.72, 21.5	0.115
cardiogenic_shock	76	5.47	0.66, 45.0	0.114
DM	76	1.48	0.35, 6.22	0.589
MI	76	2.37	0.48, 11.7	0.291
PCI	76	1.62	0.33, 8.05	0.552
CABG	76	2.50	0.50, 12.4	0.262
AO	76	2.63	0.53, 13.0	0.236
udar	76	1.20	0.15, 9.76	0.865
Smoking	76	5.39	1.09, 26.7	0.039
Chronic kidney disease	76	3.58	0.85, 15.0	0.082
COPD	76	1.63	0.20, 13.2	0.648
medicine_clopidogrel	76	0.40	0.05, 3.26	0.393
medicine_ticagrelor	76	2.49	0.31, 20.3	0.393
medicine_betablocker	76	0.23	0.03, 1.87	0.170
medicine_Ca_blocker	76	3.17	0.76, 13.3	0.114
medicine_diuretic	76	0.86	0.17, 4.25	0.851
medicine_MRA	76	0.94	0.19, 4.66	0.939
medicine_NTG	76	1.44	0.18, 11.7	0.732
medicine_alfa_adrenolitic	76	3.82	0.91, 16.0	0.067

Characteristic	N	HR[1]	95% CI[1]	p-value
medicine_acenocumarol_warafarin	76	1.13	0.23, 5.62	0.878
medicine_IPP	76	1.46	0.18, 11.9	0.722
medicine_hypoglycaemic	76	2.65	0.66, 10.6	0.170
medicine_insulin	76	1.27	0.26, 6.31	0.768
echo_EF	67	1.04	0.96, 1.14	0.312

[1]HR = Hazard Ratio, CI = Confidence Interval
